# The effect of soil parameters and earthworm abundance on the fine‐scale nocturnal habitat use of the Eurasian woodcock (*Scolopax rusticola*)

**DOI:** 10.1002/ece3.70136

**Published:** 2024-08-06

**Authors:** Gergely Schally, Dániel Tóth, Mihály Márton, Hanna Bijl, Péter Palatitz, Sándor Csányi, Maxwell Maimela Modiba, Hanaa Tharwat Mohamed Ibrahim, Barbara Simon

**Affiliations:** ^1^ Department of Wildlife Biology and Management Institute for Wildlife Management and Nature Conservation, Hungarian University of Agriculture and Life Sciences Gödöllő Hungary; ^2^ Red‐Footed Falcon Conservation Working Group MME/BirdLife Hungary Budapest Hungary; ^3^ Department of Soil Science Institute of Environmental Sciences, Hungarian University of Agriculture and Life Sciences Gödöllő Hungary

**Keywords:** earthworm abundance, foraging, GPS telemetry, pastures, soil properties

## Abstract

The Eurasian woodcock prefers habitats where its main prey, earthworms, can be found in higher densities. Although they are forest‐dwelling birds, they regularly visit pastures and natural grasslands at night, where earthworm abundance is generally higher. However, there is little information on fine‐scale habitat use in relation to variation in habitat characteristics and prey availability, particularly beyond the breeding season. In our study, we investigated if the nocturnal occurrence of woodcocks during migratory stopover periods differed between two neighbouring fields, or management units, with similar vegetation structure, and if within‐field variation in the spatial patterns of woodcock sightings were associated with fine‐scale earthworm densities and soil parameters. Specifically, we used GPS tracking data of two tagged woodcocks and direct observation data to study patterns of occurrence of birds in a mixed forest‐pasture landscape in Hungary during pre‐ and post‐breeding periods. We compared these patterns with fine‐scale soil characteristics and earthworm abundance, acquired by field sampling. We found that the field with higher earthworm abundance was visited by woodcocks more frequently, and this correlation was similarly observed at the intra‐field level. Our results demonstrate that woodcocks select foraging sites with higher earthworm densities at multiple spatial scales, both between fields (coarse scale), and within fields (fine‐scale). Considering that woodcocks tended to return to the same field to forage at night, the strong associations between occupancy and resources provide a basis for developing habitat management strategies at the field level for conservation. As earthworm densities and soil parameters are good indicators of woodcock foraging habitat, measuring those variables, at least at a coarse scale, could aid in predicting important habitats for the species across the landscape.

## Graphical abstract

In our study, we investigated the habitat use of Eurasian woodcock in a forest‐pasture landscape by analysing soil parameters, including the bird's main prey, earthworms. We used GPS tracking and direct observation data of the birds, and we compared these patterns with fine‐scale soil characteristics acquired by field sampling. Our results confirmed that the field with higher earthworm abundance was visited by woodcocks more frequently, and this relation could also be observed even at an intra‐field level.

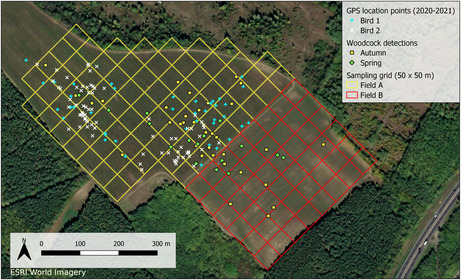

## 1 INTRODUCTION

Eurasian woodcock (*Scolopax rusticola*) is a specialist predator, with a diet comprised primarily of earthworms, sourced from both the soil surface and the upper layer of the soil profile (Hirons, 1982; Hoodless & Hirons, 2007; Kiss et al., 1989). Both the daily habitat use (Duriez, Ferrand, et al., 2005) and the annual migration of woodcocks are heavily affected by the availability of earthworms (Péron et al., 2011). Woodcocks find favourable feeding conditions both within woodland habitats and open areas (Duriez, Ferrand, et al., 2005; Duriez, Fritz, et al., 2005; Hoodless & Hirons, 2007). Diurnally, they predominantly inhabit forested and shrub‐rich areas, exhibiting relatively low activity levels. Indeed, their nocturnal behaviour is notably more active and they often move to open grasslands and pastures for foraging (Duriez, Fritz, et al., 2005; Hoodless & Hirons, 2007). Despite the elevated predation risk posed by these open areas, the higher densities of their preferred prey species necessitate woodcock to regularly visit those habitats (Duriez, Fritz, et al., 2005). Moreover, in open environments, solitary individuals may encounter conspecifics with increased frequency, facilitating opportunities for mutual assistance in foraging through information exchange on prey availability (Buxton et al., 2020). Since these birds seek out earthworm prey in open landscapes, there is an obvious connection between their habitat needs and agricultural land‐use. Understanding how woodcock use this type of landscape can allow for the development of more effective habitat management in agricultural landscapes.

As soil ecosystem engineers, earthworms mix organic and mineral constituents within the soil matrix, through their feeding and burrowing (Lavelle, 1997). These activities produce heterogeneous structures, known as earthworm casts, that provide specific physicochemical parameters to the soil (Blouin et al., 2013; Jouquet et al., 2012; Oyedele et al., 2006; Pulleman et al., 2005). Earthworms can enhance soil structural formation (Hedde et al., 2013; Ketterings et al., 1997; Mackay & Kladivko, 1985), increase the water‐holding capacity, saturated hydraulic conductivity and also the amount of water‐stable aggregates (Hallam et al., 2021; Whalley et al., 1995). These invertebrates are known to aggregate into ‘hotspots’, resulting in a heterogeneous spatial distribution (Rossi et al., 1997; van de Logt et al., 2023). Earthworms can survive in diverse habitat types, encompassing urban, agricultural, grassland and forested areas (Talavera et al., 2020), however, they usually prefer moist soil habitat (Singh et al., 2016) with high organic matter content, low soil disturbance (Dekemati et al., 2019; Ernst & Emmerling, 2009; Hoeffner et al., 2021; Lapied et al., 2009; Singh et al., 2016; Talavera et al., 2020) and soils with finer textures (Hendrix et al., 1998; Lapied et al., 2009; Singh et al., 2016). Understanding spatial associations between predator and prey at multiple spatial scales can provide important information on habitat use of woodcock in working landscapes like agroecosystems.

The seasonal changes in the availability of their prey might be the most important factor driving the annual migration cycle of the woodcocks (Berthold, 2001; Newton, 2007). In autumn, they move from Northeastern Europe and Central Siberia to Western and Southern European areas, mostly coastal, forested areas with mild and humid winter climates. Even in those places, farther southward escape migration movements can occur during cold spells (Péron et al., 2011). Conversely, they leave most of the Western and Southern European areas where the summer is relatively hot and dry, and return to their breeding grounds in spring. Knowledge from ringing and recent GPS tracking studies (Schally, 2019; Schally et al., 2022) suggest that the central European region might not be considered an area exclusively used by woodcocks for breeding or wintering. Instead, it is important during their pre‐ and postnuptial migration periods, providing stopover areas.

Pastures and natural grasslands are favourable habitats for earthworms, thus, they play an important role in the habitat use of woodcocks either at their breeding (Venetz, 2019) or wintering areas (Duriez, Ferrand, et al., 2005), and probably also at their stopovers during migration. Habitat selection can have direct effect on the success of migration, however, there is generally less information on habitat use during the migratory period. Stopover sites for this species are often agro‐forest mosaic landscapes (Crespo et al., 2016). Given the important role pastures play in food availability for woodcock, understanding habitat use within agro‐forest mosaic landscapes and the relationship with resource availability can have important implications for conservation beyond the breeding season. This is particularly true because conservation management decisions tend to be based on habitat use at larger spatial scales.

Previous studies explored the mesoscale habitat selection of the species (Crespo et al., 2016), and provided habitat‐to‐habitat comparisons (Duriez, Ferrand, et al., 2005; Hoodless & Hirons, 2007; Venetz, 2019). Our study aimed to investigate the intra‐field level microhabitat choice of woodcocks in a central European agro‐forest mosaic landscape. Fields can be regarded as management units both for agriculture and conservation (Guerrero et al., 2012), but also as distinct landscape features that could serve as important cues for the birds. Quickly locating a sufficient stopover site may determine the success of the migration (Cohen et al., 2014). To minimise the time spent with searching, the habitat choice of woodcocks might be linked to larger spatial units, such as fields at a basic level, which they may then refine based on fine‐scale characteristics. Field experience shows that within the same extensive contiguous area there can be differences in the occurrence of birds. For example, specific fields exhibit recurrent usage and year‐to‐year visitations, even if the vegetation changes. Furthermore, heterogeneity of occurrence within fields can be assumed, with a greater preference for specific patches (Colwell & Landrum, 1993).

Knowledge of land use patterns and habitat requirements of this cryptic species aids in understanding the factors influencing their spatial distribution. As a result, this knowledge contributes to the refinement of habitat suitability and species distribution models based on these factors. In our study, we used a hierarchical approach to analyse woodcock occurrence at different spatial scales (between and within field). We aimed to answer the following questions:

- Did the occurrence of woodcocks differ between two neighbouring fields that are distinct landscape features but have similar vegetation structures?
- Is there a correlation between fine‐scale earthworm densities, soil characteristics and the spatial patterns of woodcock sightings?

We anticipated that the abundance of earthworms and their aggregation in patches with favourable characteristics would be correlated with the fine‐scale nocturnal habitat use of the woodcocks.

## 2 MATERIALS AND METHODS

### 2.1 Study area

**FIGURE 1:**
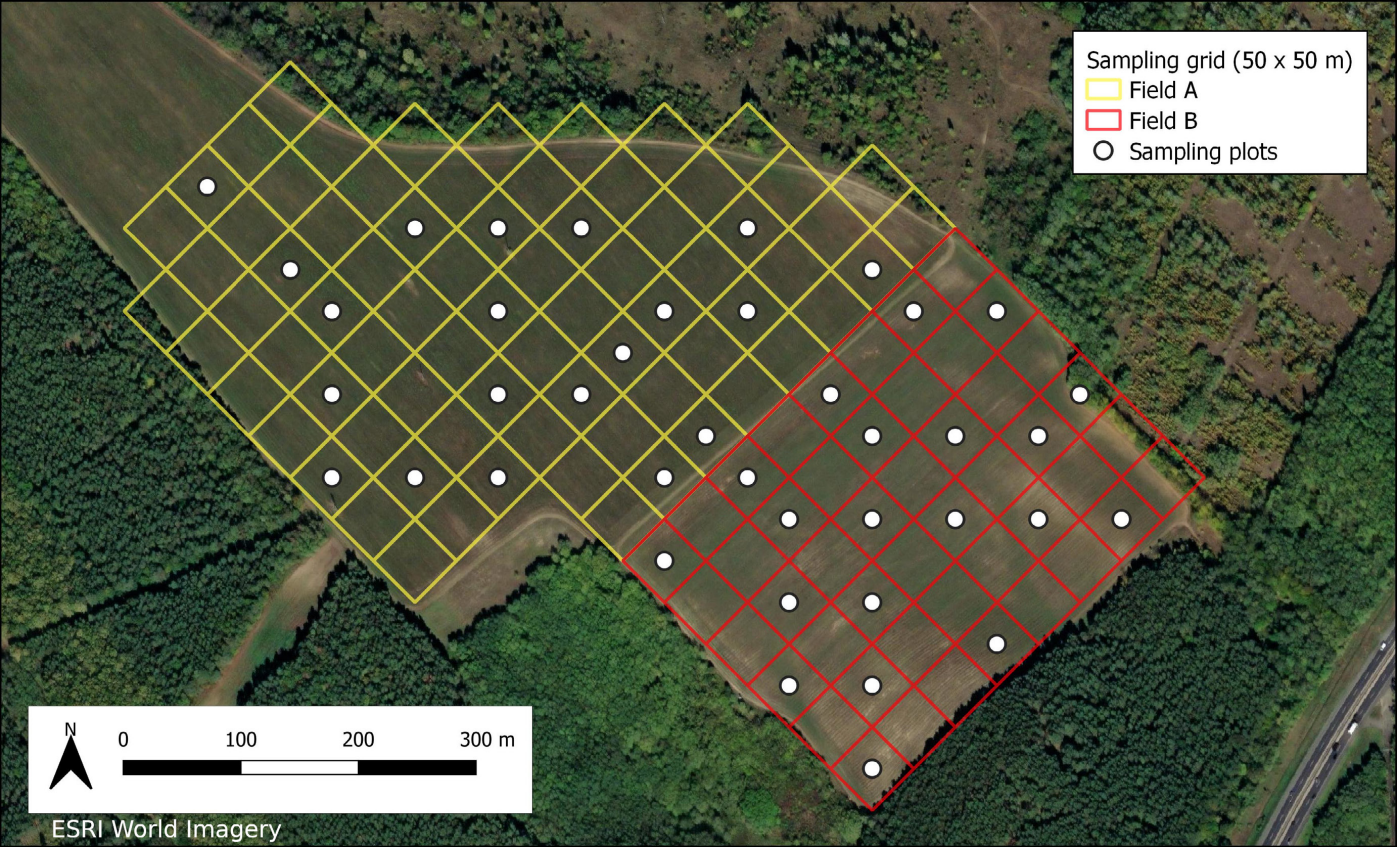
Overview of the study area and the sampling plots.

The study area is located in Gödöllő, Hungary (47.62694 N, 19.38323 E) (Figure 1). The size of the area is approximately 30 ha, surrounded by deciduous, coniferous and mixed forests. This site is used as a pasture, periodically mown for fodder intended for horses and cows. A cluster of 11 small lakes is also located in close proximity to the study area.

The study concentrated on two neighbouring fields (named ‘Field A’ and ‘Field B’), separated by a 2‐m‐wide dirt road. The vegetation structure on the two fields is similar, predominantly featuring herbaceous and grassy vegetation. Shortly before the field sampling occasions, mowing took place at the study area, therefore vegetation was characterised only post‐sampling. To evaluate the state of naturalness and degree of disturbance of the vegetation, which may be related to soil parameters and thus earthworm abundance, average cover values of five vegetation plots on each field were characterised based on Borhidi's Social Behaviour Types (Borhidi, 1995), which describe the ecological roles of species within the plant community. Both fields were dominated by plants of habitats disturbed by anthropogenic factors, yet subtle differences were still found between them. On Field A, disturbance tolerants (e.g. *Poa angustifolia*) were dominant and even competitors of natural communities (e.g. *Festuca pseudovina*) were recognised. Field B had higher cover of introduced crops (e.g. *Trifolium incarnatum* and *Medicago sativa*) and ruderal competitors (e.g. *Agropyron repens*) of natural flora. The distribution of the ecological roles indicates that the vegetation composition in the two fields is largely similar, but different stages of fallow succession can be observed.

Forested areas border both fields. Field A is 18.75 ha large and Field B has a size of 12 ha. The elevation of the study area varies between 190 and 250 m asl; the lowest parts can be found at the eastern part of Field B. The texture of the soil in the region is mostly sandy loam. According to the available land management data, in the last 5–6 years both fields were unploughed and both fields were mowed regularly.

### 2.2 Satellite tracking

The study was based on data from previous satellite tracking of woodcocks, carried out in autumn 2020 in the study area (Schally et al., 2021). For tagging, we used PinPoint GPS Argos 240 transmitters (Lotek Wireless Inc.), fitted with leg‐loop harness (Rappole & Tipton, 1991). The age of the birds was determined according to moult stages of their wing feathers (Ferrand & Gossmann, 2009). We only tagged adult (second winter or older) birds to minimise losses due to potentially high mortality in juveniles (Péron et al., 2012; Sergio et al., 2014; Tavecchia et al., 2002). The transmitters were programmed to record one GPS point daily; a nocturnal point a few minutes after midnight, and a diurnal point at noon. Only GPS location data with sufficient quality (‘G’ – ‘Ok’) were retained, based on Lotek's CRC (Cyclic Redundancy Check) quality control algorithm. Although we marked and tracked five woodcocks in the vicinity of the study area in the respective season, only the data of those two birds were used, that were actually located in the fields we examined. We tracked one of them between 7 November 2020 and 15 January 2021 (Bird 1–85 points) and the other one between 28 October 2020 and 29 March 2021 (Bird 2–179 points) inside the study area. Both birds were captured, tagged and released on Field B.

### 2.3 Collecting woodcock observation data

Observation data of the woodcocks were collected simultaneously during regular ringing fieldwork, through a nocturnal methodology involving night‐lighting (Gossmann et al., 1988). The field visits were systematically conducted to observe, catch and ring woodcocks after sunset and after complete darkness, for an average duration of 3 h. These visits were carried out eight times in autumn (October–November 2022) and four times in spring (March 2023). The same route was taken to both sites on each occasion, assuring the comprehensive coverage of both fields. This route was a single, circular transect along the outer boundaries of the two fields, maintaining a distance of 100 m from the field edges. The observation and identification of woodcocks was facilitated through handheld LED lights and binoculars. In cases of a successful capture, the specific coordinates were recorded using a GPS device. Conversely, when a woodcock was flushed, the departure location was recorded. If multiple woodcocks were detected in the same patch, the number of individuals were recorded.

### 2.4 Earthworm and soil sampling

The earthworm sampling in autumn 2022, was carried out at night, based on the assumption that certain earthworm species (mainly anecic species, e.g. *Lumbricus terrestris*) would be active and feeding in the soil surface at that time (Duriez, Ferrand, et al., 2005). Conversely, based on our experience of the autumn sampling, that no earthworm specimens, middens, or burrow openings were found on the soil surface, nocturnal sampling was deemed not to improve sampling efficiency and so spring sampling was conducted during the day only. During both occasions, we used a rectangular grid pattern to ensure adequate spatial coverage during sampling. The surveyed fields were divided into 50 × 50 m cells using geospatial tools, with 20 cells per field selected for sampling (Figure 1). The centroids of the selected sampling units were located in the field using a GPS device (Garmin Montana 750i). One soil block was then taken in each sampling unit (20 × 20 × 20 cm) using a shovel according to the ISO (International Organisation for Standardisation) 23,611–1 standard (Römbke et al., 2006).

To quantify earthworm abundance, the hand‐sorting method was used, that is, the excavated soil block was put onto a plastic sheet and was thoroughly searched for earthworms. The abundance of individuals (in. m^−2^) and their biomass (g m^−2^) were derived from the samples. Species identification based on external characteristics was accomplished according to the classification provided by Csuzdi and Zicsi (Csuzdi, 2007; Csuzdi & Zicsi, 2003). Soil samples were taken once during the study (October, 2022) to collect information about the basic soil physical and chemical characteristics. The soil samples originated from the soil blocks excavated for the earthworm sampling described above. About 150–200 g of the sampled soil was kept in a sealed and labelled plastic bag for laboratory analyses. The soil moisture content (SMC) was determined by using the gravimetric moisture determination method (Buzás, 1993) in the laboratory on the following day. Then the rest of the collected soil samples were dried, the plant residues were removed, then it was sieved through a 2‐mm sieve. Lastly, the prepared samples were then analysed for physical and chemical parameters. In total, we collected 20 (Field A) + 20 (Field B) soil samples in our experiment.

Assuming that the general patterns of soil physical (texture) and chemical parameters (soil pH, SOM) remain stable in such an undisturbed, grassy habitat without the addition of extra organic residues, manure or fertiliser added over a short period of time, soil parameters were only measured and analysed in autumn to establish background values. Consequently, biological parameters (earthworm abundance and biomass), that are more sensitive and subject to quicker changes, along with rapidly fluctuating soil parameters (e.g. SMC) were measured both in spring and autumn.

### 2.5 Laboratory processing of soil samples

#### 2.5.1 Soil physical parameters

Soil moisture content was determined by the gravimetric method in a drying oven at 105°C for 24 h (Buzás, 1993). Soil texture was determined according to Arany's method (Buzás, 1993), which involves measuring the volume of distilled water that 100 g of a soil sample can absorb until it reaches its upper plasticity limit. The more water the soil absorbs (mL of the consumed water), the more moisture it can retain, indicating a finer texture. Consequently, a higher texture value corresponds to a greater proportion of fine soil particles (clay) and increased plasticity of the sample.

#### 2.5.2 Soil chemical parameters

The soil pH was measured potentiometrically in a 1: 2.5 soil/liquid solution (either distilled water or KCl) using a pH meter (HACH‐LANGE, HQ411D) (Buzás, 1988). The soil organic carbon content (SOC) was determined by the wet oxidation method (Walkley, 1947) using concentrated H_2_SO_4_ and K_2_Cr_2_O_7_ as oxidising agents. The soil organic matter (SOM) was calculated from the obtained %SOC values, that is, it was multiplied by 1.724. The electrical conductivity of the soil samples was determined using the Jenway 4510 Conductivity Meter (Buzás, 1988). The calcium carbonate content was determined by the Scheibler method volumetrically using 10% HCl to determine the per cent CaCO_3_ content (Buzás, 1988).

### 2.6 Estimation of soil parameters for the whole study area

**FIGURE 2:**
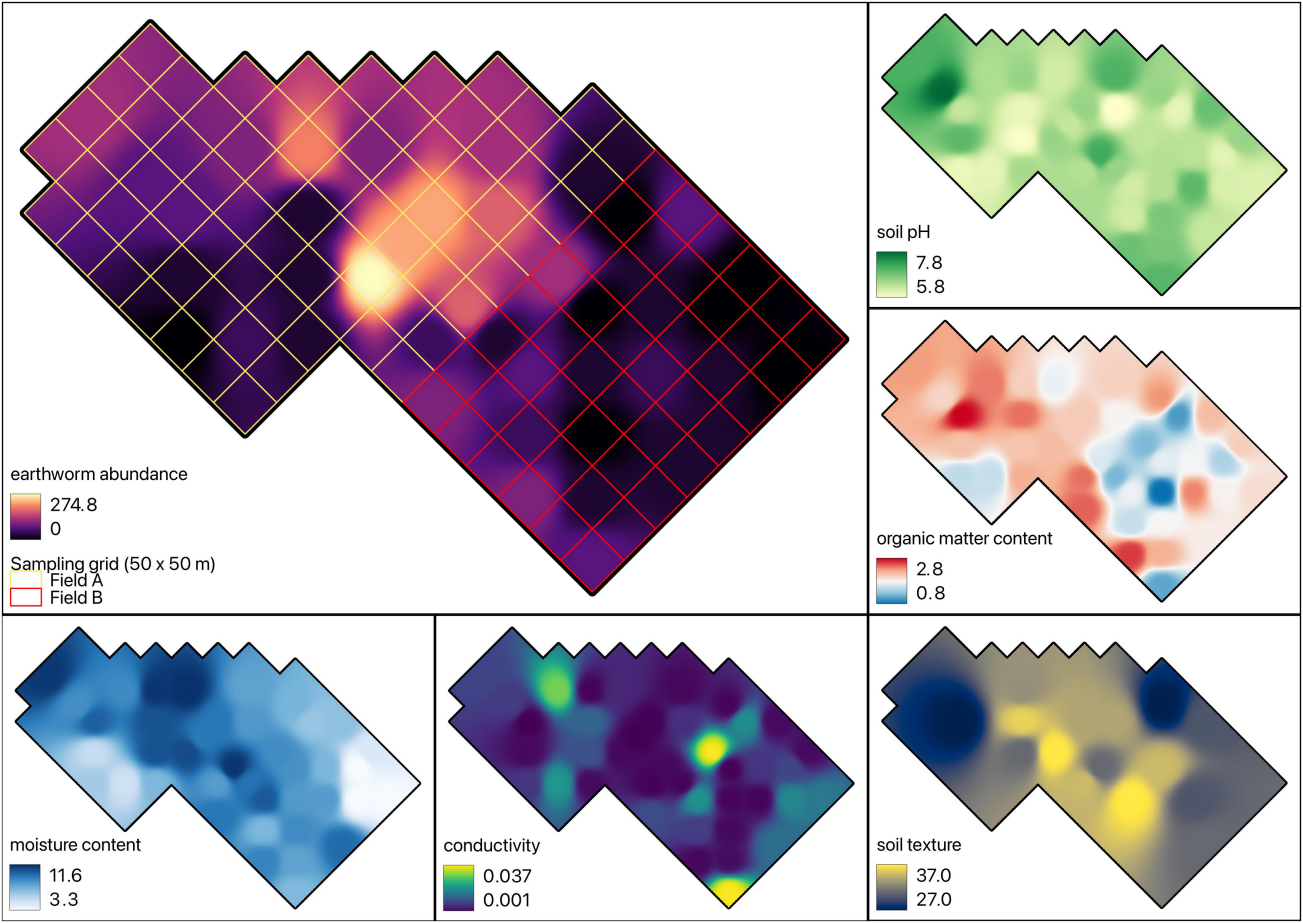
The soil attributes derived from the field sampling data in autumn.

Using the measured soil parameters for each sampling unit, a spatial interpolation method was applied to the entire study area. This interpolation used the inverse distance weighted method (Shepard, 1968) with a distance coefficient (*p* = 4) and a resolution of 1 m (Figure 2). The distance coefficient was chosen in an experimental way, by gradually increasing the value until a point where the effects of neighbouring points started to overlap. The value we chose was large enough for the cell values from field sampling affecting their neighbours, but small enough to not make hard boundaries between the two neighbouring sampling units. The sampling density of the points in our study was 1.3 points per hectare, which is regarded suitable for reliable estimations (Long et al., 2018; Radočaj et al., 2021). The values derived from interpolation were assigned to each grid based on the location of its centroid. This provided information on the soil parameters of each grid. Subsequently, the observation data of woodcocks were summarised for each grid for the spring and autumn periods based on their corresponding coordinates.

### 2.7 Data analysis

We first used the GPS tracking data to compare the occurrence of woodcocks between the two fields. Then, the statistical significance of differences in the woodcock detections registered during field observations in the two fields and between the two seasons were tested using a regular χ^2^ contingency test.

The predictive power of the collected soil parameters to distinguish between the two fields were assessed using logistic regression models. First, we applied a principal component analysis (PCA) implemented in the ‘prcomp’ function in R to eliminate collinearity and reduce the dimension of environmental variables. Then, the first two axes of the PCA which explain a significant part of variance in the original dataset, were used as explanatory variables to predict the binary response variable, which was defined as originating from either field A or B.

Following this step, the effect of each environmental variable on the field association was further investigated using the same method explained above. However, in this second analysis, all individual measurements were used during the model construction. Prior to fitting the model, all environmental variables were scaled, the collinearity between the predictors were investigated using the ‘VIF’ function in R, and a binary logistic regression model was fitted to the data in a backward step selection.

The predictive power of the collected soil parameters within fields were evaluated using the same method explained above, by analysing the correlation between soil parameters collected from sample grids and the response variable, which was defined as at least one instance of visual observation of a woodcock (positive) or non‐observation (negative). Only field observation data were used for the comparison, and no GPS tracking data were included.

Finally, earthworm abundances were compared between fields and between positive and non‐detection grids within fields both in autumn and in spring, and between seasons within fields using Mann–Whitney U‐test.

Geospatial analyses and data mapping were performed using Quantum GIS (v3.30), and all additional statistical analyses were performed using PAST (v4.13) as well as the packages ‘car’ and ‘pscl’ implemented in R software version 4.3.2 (R Core Team, 2023).

## 3 RESULTS

### 3.1 Differences in woodcock observations between the fields

**FIGURE 3:**
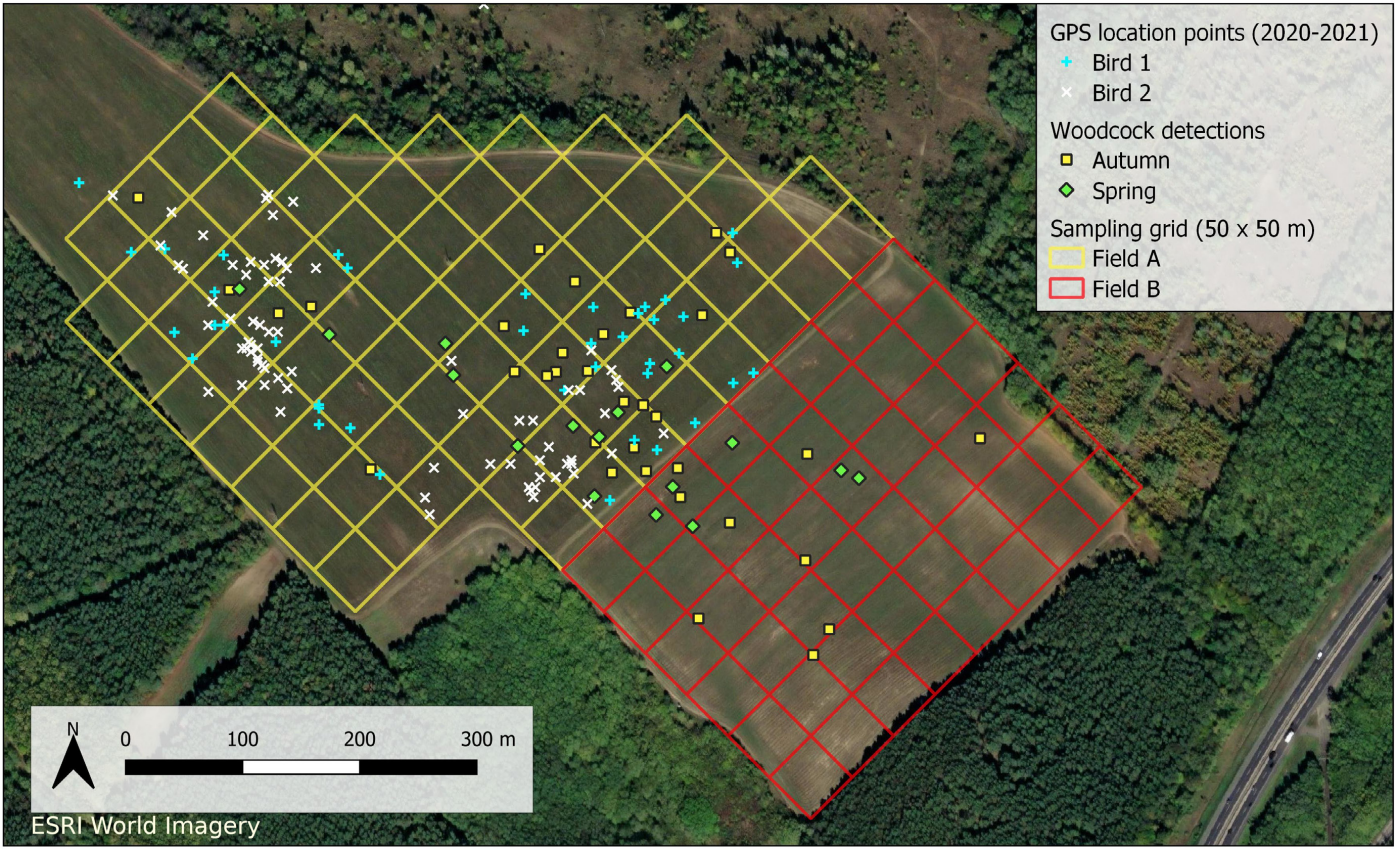
Distribution of woodcock observations and GPS locations in the two fields.

We found difference in the total number of woodcock observations recorded between the two fields in spring and autumn (Figure 3). Field A had two to three times higher number of woodcock observations in total (Autumn: *n* = 28; Spring: *n* = 11) than Field B (Autumn: *n* = 9; Spring: *n* = 6), and the density of the observations was also higher on Field A (Autumn: 0.37 observation/grid; Spring: 0.15 observation/grid) than on Field B (Autumn: 0.19 observation/grid; Spring: 0.13 observation/grid). The Chi‐square contingency test for field and season indicated that there was no difference in the observations among field‐season combinations (χ^2^_1_ = 0.699; *p* = .40). The woodcocks tracked in autumn 2020 frequently moved from their diurnal forest shelters to feed in the study area at night (Bird 1: 40/64 nights – 63%; Bird 2: 76/147 nights – 52%), but both woodcocks exclusively used Field A and never visited Field B (Figure 3).

### 3.2 Differences in soil and earthworm parameters between the fields

To identify differences between fields, we modelled field association as the response variable. PCA results show that more than 62% of the variation in the original environmental variables (earthworm abundance, soil pH, SMC, SOM, texture and electrical conductivity) were explained by the first two axes (Table 1). We have found a statistically significant correlation between PCA transformed environmental variables and the response variable with an estimated coefficient of 0.59 (*p* = .01).

**TABLE 1:** Principal component analysis (PCA) results of the environmental variables collected in autumn.

	Variance explained (%)	Component					
		PC 1	PC 2	PC 3	PC 4	PC 5	PC 6
		35.29	27.01	14.96	11.43	6.05	5.27
Loading estimates	Soil pH	0.50	−0.64	0.30	−0.40	0.02	0.30
	Soil texture	0.17	0.89	−0.19	−0.01	0.05	0.38
	EW abundance	0.80	0.35	0.18	−0.07	−0.44	−0.14
	SOM	0.59	−0.42	−0.13	0.67	−0.02	0.14
	SMC	0.81	0.29	0.22	−0.02	0.41	−0.19
	Electrical conductivity	−0.45	0.22	0.82	0.27	−0.02	0.08

Abbreviations: EW, earthworm; SMC, soil moisture content; SOM, soil organic matter.

The stepwise model selection identified a model with a subset of five explanatory variables, which were earthworm abundance, soil pH, SOM, texture and electrical conductivity as the best fit model since this model had the lowest AIC (see Supporting information for a list of models) and a negligible level of multicollinearity (1.77–4.98) between variables were observed. The field association, as a response variable, was positively correlated with earthworm abundance and SOM, but negatively with soil pH, texture and electrical conductivity. The highest effect size was found in the case of earthworm abundance (Table 2). In general, the regression model was highly significant and the model explained approximately 60% of variation within the data (McFadden's R^2^ = 0.58; χ^2^_5_ = 95.5; *p* < .001).

**TABLE 2:** Output of the logistic regression model comparing the environmental variables among fields in autumn.

	Standardised estimate	Std. error	*z* value	*p* value
(Intercept)	1.49	0.48	3.09	.002
Soil pH	−2.26	0.64	−3.55	<.001
Texture	−2.63	0.65	−4.06	<.001
EW abundance – autumn (in. m^−2^)	4.22	0.93	4.53	<.001
SOM (%)	1.11	0.44	2.50	.01
Electrical conductivity (μS/cm)	−0.78	0.47	−1.67	.10

Abbreviations: EW, earthworm; SOM, soil organic matter.

The following earthworm species were found during field sampling: Autumn: *Aporrectodea rosea* (Fields A and B), *A. caliginosa* (Fields A and B) and *A. trapeziodes* (Field A); Spring: *A. rosea* (Fields A and B) and *A. caliginosa* (Fields A and B). *A. rosea* was the most common species both in autumn and in spring (50% and 64% of all identified individuals), *A. caliginosa* was also present in both seasons (31% and 36%). In comparison, *A. trapezoides* was found only in autumn (19% of all identified individuals). Significant differences in earthworm abundance were found between the two fields both in autumn (U = 608; *p* < .001) and in spring (U = 475; *p* < .001). The abundance of earthworms in Field A (Autumn: 100.7 in. m^−2^; Spring: 103.3 in. m^−2^) was more than twice as much as in Field B (Autumn: 38.5 in. m^−2^; Spring: 46.4 in. m^−2^) (Table 3). The large standard deviation indicates a high heterogeneity within the fields. No seasonal difference was found in earthworm abundance within fields (Field A: U = 2753.5; *p* = .83; Field B: U = 965.5; *p* = .17). Earthworms were present in the majority of the sampling plots. However, there was a remarkable difference in the proportion of the sampling plots with no earthworm detections: Autumn – Field A: 5%, Field B: 30%; Spring – Field A: 5%, Field B: 20%. The average biomass of earthworms was 19.5 g m^−2^ (SD = 15.77) in autumn and 22.0 g m^−2^ (SD = 16.07) in spring for the whole study area.

**TABLE 3:** Soil and earthworm parameters of the sampled fields.

Parameter	Season	Field A			Field B		
		Mean	Median	SD	Mean	Median	SD
SMC (%)	Autumn	9.0	9.4	1.6	7.4	8.1	2.0
Texture	Autumn	31.5	32.2	2.7	32.4	31.4	2.3
Soil pH	Autumn	6.6	6.5	0.4	6.5	6.5	0.3
SOM (%)	Autumn	2.1	2.1	0.3	1.8	1.8	0.4
Electrical conductivity (μS/cm)	Autumn	0.007	0.010	0.007	0.010	0.010	0.009
EW abundance (in. m^−2^)	Autumn	100.7	100.4	57.4	38.5	27.3	30.3
EW abundance (in. m^−2^)	Spring	103.3	100.0	48.0	46.4	49.8	32.1
EW biomass (g m^−2^)	Autumn	26.3	26.6	15.2	9.1	5.7	9.8
EW biomass (g m^−2^)	Spring	29.8	27.3	15.0	9.8	8.9	8.1

Abbreviations: EW, earthworm; SMC, soil moisture content; SOM, soil organic matter.

### 3.3 Relationships between soil parameters and the probability of woodcock observations

We found a statistically significant correlation between the PCA‐transformed environmental variables represented by the first two axes and the woodcock detections as a response variable with an estimated coefficient of −1.48 (*p* < .001).

Stepwise model selection reported the lowest AIC value for a model that had earthworm abundance, SOM and electrical conductivity as explanatory variables (S1). No significant level of multicollinearity between variables were observed (1.12–1.14). According to the results of the logistic regression model statistically significant associations were found between woodcock detections and soil parameters (McFadden's R^2^ = 0.17; χ^2^_3_ = 21.21; *p* < .001). Woodcock detections were positively associated with earthworm abundance, but negatively associated with SOM and electrical conductivity (Table 4).

**TABLE 4:** Output of the logistic regression model comparing the environmental variables to woodcock detections in autumn.

	Standardised estimate	Std. error	*z* value	*p* value
(Intercept)	−1.60	0.27	−5.84	<.001
EW abundance – autumn (in. m^−2^)	0.62	0.23	2.65	.01
SOM (%)	−0.65	0.28	−2.33	.02
Electrical conductivity (μS/cm)	−0.83	0.32	−2.58	.01

Abbreviations: EW, earthworm; SOM, soil organic matter.

While statistically significant difference in earthworm abundance was found between the two types in autumn (U = 917; *p* < .05), there was no statistical difference in spring (U = 706.5; *p* = .66). On average, the abundance of earthworms in positive sites was more than one and a half times higher than in non‐detection areas in autumn (positive: 106.9 in. m^−2^; non‐detection: 68.3 in. m^−2^), compared to that of spring (positive: 88.4 in. m^−2^; non‐detection: 80.1 in. m^−2^) (Table 5).

**TABLE 5:** Soil parameters of the sampling sites with (Positive) or without (Non‐detection) woodcock detection records.

Parameter	Season	Positive			Non‐detection		
		Mean	Median	SD	Mean	Median	SD
SMC (%)	Autumn	9.1	9.5	1.8	8.2	8.3	1.9
Texture	Autumn	33.0	34.2	2.8	31.6	31.4	2.5
Soil pH	Autumn	6.5	6.5	0.3	6.5	6.5	0.4
SOM (%)	Autumn	2.0	2.0	0.3	1.9	2.0	0.4
Electrical conductivity (μS/cm)	Autumn	0.009	0.01	0.008	0.004	0.01	0.006
EW abundance (in. m^−2^)	Autumn	106.9	93.2	78.4	68.3	55.5	47.4
EW abundance (in. m^−2^)	Spring	88.4	71.0	53.0	80.1	75.0	50.6
EW biomass (g m^−2^)	Autumn	25.2	26.1	19.6	18.0	14.7	14.3
EW biomass (g m^−2^)	Spring	21.8	18.8	13.1	22.0	19.6	16.5

Abbreviations: EW, earthworm; SMC, soil moisture content; SOM, soil organic matter.

## 4 DISCUSSION

### 4.1 Differences in woodcock observations between the fields

We found associations between woodcock presence and earthworm abundance both at the between‐ and within field scale in a central European agro‐forest mosaic landscape. Given the significant difference in bird occurrence between the two adjacent and superficially similar study fields, this study demonstrates a preference for higher earthworm abundance and certain soil parameters that is not apparent from measures of vegetation structure alone. This preference is substantiated by observation data collected during autumn and spring, and corroborated by satellite tracking data. These results are in accordance with previous studies confirming the relationship between earthworm abundance and several bird species, for example, robin (*Erithacus rubecula*), blackbird (*Turdus merula*), fieldfare (*Turdus pilaris*) and song thrush (*Turdus philomelos*) (Martay & Pearce‐Higgins, 2020). Although visual detections were still recorded on Field B, their numbers were much lower in both seasons. GPS locations of the tracked birds, which both were captured on Field B, were later exclusively recorded on Field A. The tags of the tracked birds were programmed with the priority of a migration study, so there was only one location point at a fixed time for each night. Consequently, we cannot entirely exclude the presence of the tagged woodcocks in Field B during their whole foraging trip. However, as the tagged birds were both adults, they might already have developed a detailed spatial memory map of the landscape, and tried to maximise their foraging effectiveness by limiting their movements to patches associated with higher prey density (Colwell & Landrum, 1993).

The success of the woodcocks in finding earthworms might depend on the combination of prey detection at a fine scale (favourable patches) and a spatial memory at the landscape scale (favourable fields). The ability of birds to find earthworms through indirect cues can be particularly important for them in periods or areas of food scarcity. The constant, active search for food is not only energy intensive, but can also greatly increase the risk of predation (Duriez, Fritz, et al., 2005). Therefore, the important question is what information birds use to find the fields and areas that are the most suitable to them. Woodcocks use their bills to probe the soil surface while feeding, facilitated by special mechanoreceptors called as ‘Herbst corpuscule’ (du Toit et al., 2022). This phenomenon referred to as ‘remote touch’ involves the recognition of subterranean prey through the perception of high‐frequency acceleration components within mechanical vibrations originating from the soil outside the bill's vicinity (Gottschaldt, 1985). These vibrations are derived from the movement of the prey within the substrate. Thus, this allows woodcocks to effectively find higher earthworm densities, not only at a field level, but also at a finer scale within the feeding habitat. Moreover, specific soil parameters, for example, water content (du Toit et al., 2024) can influence the propagation of these mechanical vibrations within the soil, thereby helping birds to locate their prey.

### 4.2 Soil characteristics and earthworm distribution

Based on the individual soil parameters, neither Field A nor Field B can be considered an ideal habitat for earthworms, or at least there might be other habitats that provide much more preferable conditions. Earthworm abundance in the study area, where the soil texture was sandy loam, slightly lower abundances were found than that reported in other studies (Barras et al., 2022; Ernst & Emmerling, 2009; Nuutinen et al., 1998; Onrust & Piersma, 2019). However, in addition to abundance, the vertical distribution and surface activity of the prey affects their availability for the birds (Onrust & Piersma, 2017).

The abundance of earthworms was significantly higher in Field A than in Field B. Notably, the species *A. trapezoides* was only found in Field A and not in Field B, and only in autumn. The spatial occurrence of earthworms was heterogeneous in both fields, but the patches with relatively high densities were found in Field A. It is important to note that during the sample collection, earthworms were almost exclusively found in the 0–5 cm layer of soil among the overlying vegetation's roots. In the deeper layers, no worms were observed nor any burrows indicating their presence. This phenomenon is probably beneficial for woodcocks, given that the worms are concentrated in a zone easily accessible to birds with a bill length of approximately 70 mm. The nocturnal autumn sampling aimed at investigating species that are near the ground surface only at night (Duriez, Fritz, et al., 2005); however, such species were not encountered during the study. According to van de Logt et al. (2023), several reasons may contribute to this: first, soils with low clay contents might be disadvantageous for deep‐burrowing species, such as *Lumbricus terrestris* due to their susceptibility to drought. Second, *Lumbricus terrestris* might also be limited by interspecific competition, as they cannot match the faster growth and reproduction rate of smaller earthworm species (Butt, 1998). Additionally, anecic earthworms might be more sensitive to any kind of soil disturbance (Lindahl et al., 2009). While the earthworm species that we found by field sampling provided sufficient feeding opportunities for the woodcocks, the low diversity of earthworm species and the lack of epigeic and anecic species suggests that habitat quality could still be improved in the area.

Both SOM and SMC were slightly higher in Field A. The reason why the lower‐lying Field B was found to be slightly drier could be that the declination is more pronounced and directed southward there; thus, it was more exposed to the drying effect of the sun from autumn to spring. However, no differences in these parameters could explain the substantial variations in the abundance of earthworms.

Soil texture, SMC and electrical conductivity were slightly higher in the positive sample areas; however, the differences were so small that they should not significantly affect earthworms' occurrence or detectability. Additionally, soil humidity plays a key role in the survival and activity of earthworms (Satchell, 1967). The average moisture content, for example, was not only significantly lower than the optimal 60%–70% range found in the Jura Mountain (Venetz, 2019), but it was even lower than the values in the range of 9%–40% reported in previous studies (Barras et al., 2022; Dekemati et al., 2019; Lapied et al., 2009). Similarly, the pH values and organic matter content were within a narrow range. Therefore, it cannot be excluded that greater differences might actually influence the birds' choice. Most earthworm species occur in the pH range 5.0–7.4 (Satchell, 1967), thus all our sampling plots might fall in an optimal range in this regard.

The soil texture of the studied fields was sandy loam. According to previous studies, earthworm presence correlates with clay and stilt content, due to compaction and/or waterlogging (Curry, 2004; Lapied et al., 2009; van de Logt et al., 2023). Conversely, sandy soil, typically characterised by low water retention, generally does not provide suitable conditions for earthworms. However, these soils may be favourable for woodcock foraging, as they can feed more easily with their bills in the looser sandy soil. Additionally, sandy soils tend to more quickly warm up and exhibit more resistance to winter freezing than more densely compacted, clay soils (Abu‐Hamdeh & Reeder, 2000), thus they can provide feeding opportunity, for example, for the migrating birds in early spring.

## 5 CONCLUSIONS

Earthworms are the most widespread soil ecosystem engineers in temperate ecosystems (Blouin et al., 2013), thus their specialist predator, the woodcock, is a decent indicator of ecosystem‐level health, at least in terms of the below‐ground ecosystem. This study showed that woodcocks could adjust their feeding area based on fine‐scale earthworm abundance (and probably availability), with higher earthworm abundance correlating with areas where woodcocks were more frequently observed actively feeding. Based on these findings, we conclude that surface cover alone does not provide sufficient information to assess the suitability of habitats for woodcock in the pre‐ or post‐breeding period; other factors, especially moisture, texture and organic matter content, should also be considered.

As earthworm densities and soil parameters are good indicators of woodcock foraging habitat, measuring those variables, at least at a coarse scale, could aid predicting important habitats for the species across the landscape. While extensive fine‐scale sampling of these parameters would be difficult, it would be still possible to organise field‐level sampling to understand which general areas may be important for the birds. This information would be relevant for the conservation and management of woodcocks in their stopover areas which play critical role in the success of their migration.

This, together with the site fidelity of woodcocks within their home range, may also offer habitat management options for conservation. However, in the same region, areas rich in food (e.g. shelter lanes, non‐cultivated field margins of agri‐environmental schemes) and game fields used in wildlife management are usually characterised by tall vegetation. Sustainable management should consider that, ideally, undisturbed soil with low vegetation (Pearce‐Higgins & Yalden, 2003) and rich biodiversity may provide the necessary feeding habitat for woodcocks. Historically, large areas with low‐intensity grazing and set asides provided suitable habitats for birds feeding on soil‐dwelling invertebrates (e.g. the Northern lapwing *Vanellus vanellus*, or the Eurasian curlew *Numenius arquata*) or insectivore game species (e.g. the grey partridge *Perdix perdix*) in the region (Báldi et al., 2005). Therefore, the ongoing intensification of agriculture necessitates to develop management methods that promote soil‐dwelling invertebrates, to re‐establish the sustainable conservation and management of our landscape diversity. Such methods include perennial land use and no‐till farming (Lindahl et al., 2009), as well as organic fertilising, that is, manure (Onrust & Piersma, 2019).

Moving forward, our study was confined to a single study area, and a short period of time. To effectively identify extreme instances within soil parameter profiles, and to draw broader conclusions, it is advisable to replicate the study across multiple areas with different soil characteristics as well as in different seasons. This could help inform management policies not only beyond the small region in which this study was conducted, but also for different seasons beyond what was addressed here.

## AUTHOR CONTRIBUTIONS

**Gergely Schally:** Conceptualization (lead); data curation (lead); formal analysis (lead); investigation (equal); methodology (equal); supervision (equal); visualization (lead); writing – original draft (lead); writing – review and editing (equal). **Dániel Tóth:** Conceptualization (equal); investigation (equal); writing – review and editing (supporting). **Mihály Márton:** Conceptualization (equal); methodology (equal); writing – review and editing (supporting). **Hanna Bijl:** Writing – original draft (equal); writing – review and editing (supporting). **Péter Palatitz:** Investigation (equal); writing – review and editing (equal). **Sándor Csányi:** Supervision (equal); writing – original draft (equal); writing – review and editing (equal). **Maxwell Maimela Modiba:** Investigation (equal); writing – review and editing (supporting). **Hanaa Tharwat Mohamed Ibrahim:** Investigation (equal); writing – review and editing (supporting). **Barbara Simon:** Conceptualization (equal); investigation (equal); methodology (lead); supervision (equal); writing – original draft (equal); writing – review and editing (equal).

## CONFLICT OF INTEREST STATEMENT

The authors have no relevant financial or non‐financial interests to disclose.

## Supporting information


Appendix S1.

## Data Availability

Data availability statement The data that support the findings of this study are available in the supplementary material of this article.
